# Reporting a Homozygous Case of Neurodevelopmental Disorder Associated With a Novel *PRPF8* Variant

**DOI:** 10.1002/mgg3.70084

**Published:** 2025-03-11

**Authors:** Mohammad Reza Mirinezhad, Farzaneh Mirzaei, Arash Salmaninejad, Reza Jafarzadeh Esfehani, Mohammad Reza Seyedtaghia, Sheyda Farahmand, Mehran Beiraghi Toosi, Somayyeh Hashemian, M. E. Suzzane Lewis

**Affiliations:** ^1^ Department of Medical Genetics Faculty of Medicine, Mashhad University of Medical Sciences Mashhad Iran; ^2^ Blood Borne Infections Research Center, Academic Center for Education, Culture & Research (ACECR) Razavi Khorasan Branch Mashhad Iran; ^3^ Department of Medical Genetics, Faculty of Medicine Hormozgan University of Medical Sciences Bandar Abbas Iran; ^4^ Department of Biology Mashhad Branch, Islamic Azad University Mashhad Iran; ^5^ Pediatric Ward School of Medicine, Mashhad University of Medical Sciences Mashhad Iran; ^6^ Pediatric Neurology Research Center Mashhad University of Medical Sciences Mashhad Iran; ^7^ Mashhad University of Medical Sciences Mashhad Iran; ^8^ Department of Medical Genetics University of British Columbia (UBC) Vancouver British Columbia Canada; ^9^ BC Children's Hospital Research Institute Vancouver British Columbia Canada

**Keywords:** autism spectrum disorder, intellectual disability, neurodevelopmental disabilities, retinitis pigmentosa, spliceosome

## Abstract

**Background:**

While recently identified heterozygous *PRPF8* variants have been linked to various human diseases, their role in neurodevelopmental disorders (NDDs) remains ambiguous. This study investigates the potential association between homozygous *PRPF8* variants and NDDs. Most *PRPF8* variants are primarily associated with retinal diseases; however, we analyze a family with multiple members diagnosed with NDDs.

**Methods:**

Using exome sequencing (ES), the cause of behavioral problems and intellectual disabilities (IDs) of two sisters from a consanguineous parents was solved, and the results confirmed by direct sanger sequencing method likewise protein modeling to assess the structural impact of the identified variant on the PRPF8 protein has been done.

**Results:**

ES identified a novel homozygous variant, PRPF8 c.257G>T, p.R86M. To the best of our knowledge at the time of writing this manuscript, the mentioned variant has not been reported in relation to NDDs. Protein modeling provided another line of evidence proving the pathogenicity of the novel variant.

**Conclusion:**

Our findings indicate that the p.R86M variant may disrupt normal protein function by changing its structure and probably its interaction, potentially leading to the observed neurodevelopmental phenotypes. This study highlights the first link between the *PRPF8* variant and NDDs, suggesting a distinct role for specific *PRPF8* variants in the etiology of NDDs. These results warrant further investigation into the mechanisms by which *PRPF8* variants contribute to NDDs, emphasizing the need for comprehensive genetic screening in families with unexplained neurodevelopmental conditions.

## Graphical abstract

First report of a homozygous case of neurodevelopmental disorder associated with a novel *PRPF8* variant. A family identified with a novel homozygous variant, PRPF8 c.257G>T, p.R86M diagnosed with NDDs.

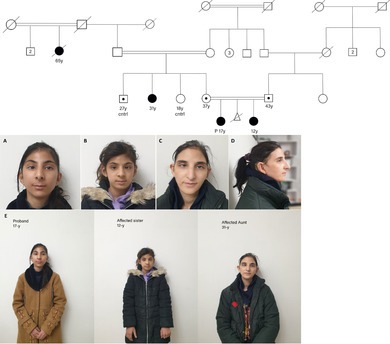

## 1 Introduction

Neurodevelopmental disorders (NDDs) encompass a heterogeneous group of conditions characterized by atypical development and functioning of the central nervous system (Servetti et al. 2021). These disorders manifest during the early stages of brain development and often persist throughout life, impacting various domains, including cognition, communication, social interaction, and motor skills (Morris‐Rosendahl and Crocq 2020). While NDDs include a wide range of disorders such as autism spectrum disorder, intellectual disability (ID), and attention‐deficit/hyperactivity disorder, their etiology is complex and likely involves a combination of genetic and environmental factors (Antolini and Colizzi 2023). Although the specific mechanisms underlying each disorder are still being elucidated, research suggests that disruptions in neurodevelopmental pathways involving neuronal migration, synaptogenesis, and myelination might play an important role in the development of NDDs (Kroon et al. 2013).

During the complex process of gene expression, the pre‐mRNA processing factor 8 (*PRPF8*) gene plays a pivotal role as a crucial component of the splicing machinery (Xia et al. 2021). This gene exerts a profound influence on the fidelity and efficiency of pre‐mRNA splicing, a fundamental process that converts pre‐mRNA transcripts into mature, protein‐coding mRNAs. The role of *PRPF8* extends beyond its immediate function in splicing; also, remarkable conservation across eukaryotic species highlights its fundamental importance in development. However, mutations within the *PRPF8* gene have been linked to various human pathologies, suggesting its involvement in maintaining cellular homeostasis and normal development (Grainger and Beggs 2005). Disruptions in protein production, orchestrated by faulty splicing due to *PRPF8* mutations, manifest as human diseases affecting various organs. In the ocular system, this can lead to autosomal dominant retinitis pigmentosa (adRP), a progressive neurodegenerative disease‐causing photoreceptor cell death and subsequent vision loss (Liu et al. 2022). Similarly, mutations in *PRPF8* have been implicated in the development of myeloid malignancies, like acute myeloid leukemia (AML) and myelodysplastic syndromes, by disrupting splicing in blood progenitor cells and promoting uncontrolled proliferation (Kurtovic‐Kozaric et al. 2015).

Recent studies have proposed PRPF8 as a candidate gene associated with developmental delay and autism spectrum disorder (ASD). A bioinformatic analysis of monogenic ID identified PRPF8 as a key hub in gene‐interaction networks linked to autism, epilepsy, and facial dysmorphisms (Casanova et al. 2018). A de novo missense variant of uncertain significance in PRPF8 was also discovered in a child with ASD within a Brazilian cohort (da Silva Montenegro et al. 2020; Grainger and Beggs 2005). Additionally, there is an enrichment of PRPF8 sequence variants in individuals with NDDs compared to normal controls, as observed in a large sequencing database (da Silva Montenegro et al. 2020; Karczewski et al. 2020). Moreover, emerging clinical and case report evidence suggests a potential link between PRPF8 mutations and NDDs such as ASD and ID (O'Grady et al. 2022). While the exact mechanisms remain under investigation, the potential role of altered splicing in genes crucial for brain development and function is actively being explored.

Regarding the recent line of evidence suggesting a novel role for *PRPF8* variants in the development of NDDs, we report a consanguineous family with multiple individuals affected by similar syndromic features and NDDs who carry a homozygous missense variant in the *PRPF8* gene at residue 86: NP_006436.3.

## 2 Material and Methods

### 2.1 Ethical Compliance

This study was conducted in accordance with the Declaration of Helsinki. Participants provided informed and voluntary consent for the publication of their information and images and signed a written consent form. For individuals under the age of 18, consent was also obtained from their legal guardians.

### 2.2 Exome Sequencing and Data Analysis

Exome Sequencing (ES) was used to enrich all exons of protein‐coding genes using the Agilent SureSelect V7 on qualified gDNA randomly sheared by the ultrasonic high‐performance sample processing system (Covaris). Next‐generation sequencing (NGS) was conducted, generating close to 100 million reads on an Illumina MiSeq Sequencer. In general, the test platform examined > 95% of the targeted regions with sensitivity exceeding 99%. The mapping of reads was performed using BWA‐MEM with the hg19 genome assembly, and variant calling was conducted with HaplotypeCaller (GATK, 4.0.2). In this test, point mutations and microinsertion/deletions and duplication (< 20 bp) can be simultaneously detected.

The analysis of variant lists in Excel was conducted by filtering variants with a frequency of < 1% and focusing on pathogenic and likely pathogenic variants in the primary genes list (PGL), prioritizing homozygous variants based on the pedigree under investigation; however, this approach did not yield satisfactory results. Subsequently, employing broader criteria, the patient's data were independently analyzed in Excel by three experienced individuals who discussed the final candidate variants. Ultimately, the selected variants were reported based on a comprehensive consideration of the conditions associated with each variant.

Bioinformatics analysis of the sequencing results was carried out using international databases (Franklin, Varsome) and standard bioinformatics software (Varaft). Variants reported by ES were interpreted according to American College of Medical Genetics (ACMG) guidelines for interpreting the pathogenicity of genetic variants (Richards et al. 2015). Specific primer pairs were designed for candidate variants by using the primer3 software, and Sanger sequencing of the variant was performed by 3500 Genetic Analyzer.

The analysis of CNVs was also conducted based on the ES data using the ExomeDepth pipeline (Plagnol et al. 2012); however, none of the identified variants that could potentially explain the phenotype were confirmed.

### 2.3 Protein Modeling

MoDAFold method was employed to investigate the effect of the missense variant (Zheng et al. 2024). To achieve this, we first modeled the wild‐type (NP_006436.3) and the mutated proteins using the AlphaFold server (https://deepmind.google/technologies/alphafold/), followed by energy minimization of both structures by ModRefiner. Finally, we compared the two models using TM score and RMSD of the two models (Xu and Zhang 2011).

## 3 Case Description and Results

**FIGURE 1:**
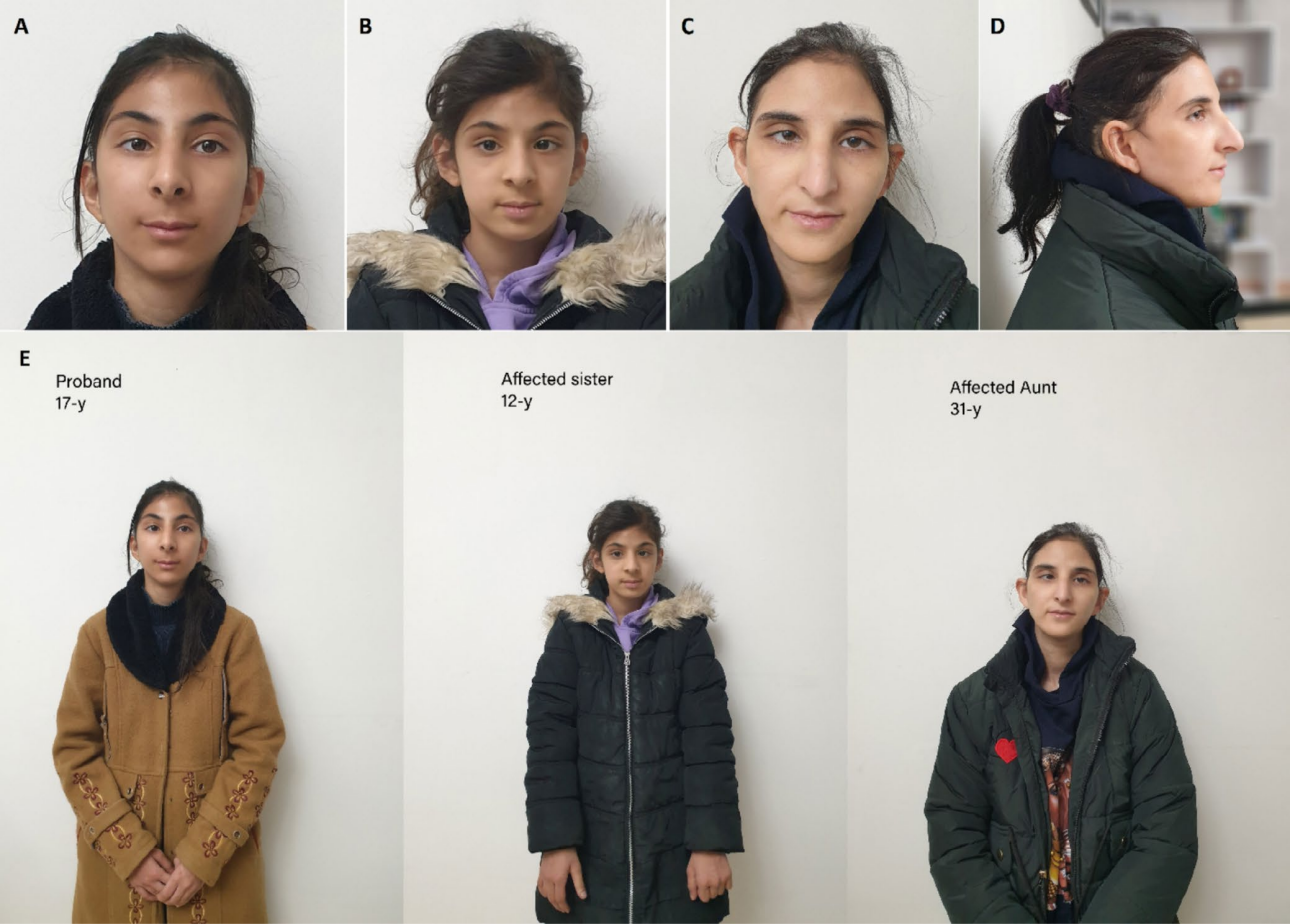
The clinical phenotype of (A) proband, (B) affected sister, (C, D) affected aunt, (E) affected family members.

A 17‐year‐old female and her 12‐year‐old sister were referred for genetic testing due to behavioral problems and ID. These sisters were offspring of consanguineous parents (first cousin marriage), with both siblings showing similar clinical manifestations (Figure 1). Both girls were born following a normal term delivery. At birth, both were noted to be small for gestational age and presented with microcephaly.

Developmentally, starting at age 3 years, both sisters exhibited language delays, intellectual deficits, and progressive behavioral issues, including restricted repetitive behaviors, hyperactivity and impulsivity, emotional dysregulation, poor impulse control, and deficits in social communication and interaction, which hindered both sisters from completing their education in elementary school. Clinical diagnosis of ASD was made according to DSM‐5 criteria at the time of the visit, as both girls experienced hypersensory challenges (particularly sensitivity to touch) and exhibited repetitive movements (flapping hands), difficulty making friends or initiating conversations, deficits in appropriately expressing emotions, and troubles understanding body language.

On physical examination, the predominant findings were microcephaly and short stature. The patients' weight and height were both less than the third centile for their age. They exhibited syndromic craniofacial dysmorphisms characterized by a beaked nose, long alar cartilage, receding forehead, prominent eyes, wide‐spaced teeth, well‐defined arched eyebrows, and long eyelashes. Neither sister had any neurological deficits, such as hearing loss, deafness, seizures, or vision problems. The two sisters were candidates for exome sequencing, as previous high‐resolution karyotyping for both had yielded unremarkable results.

**FIGURE 2:**
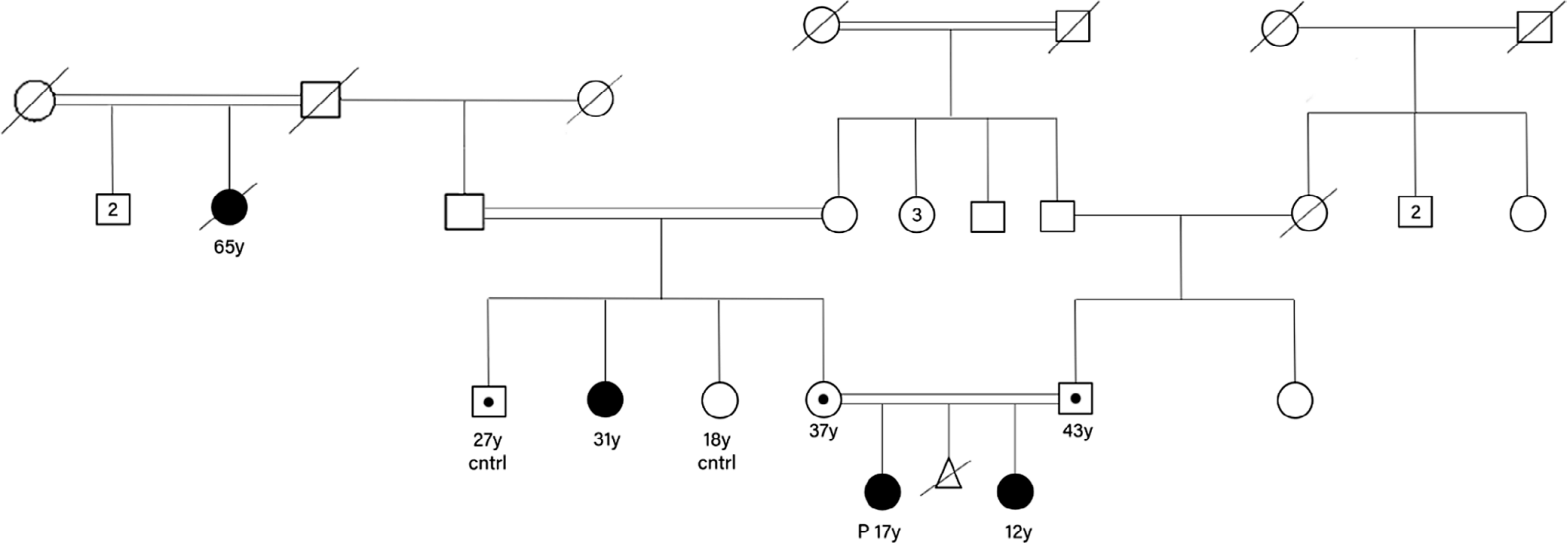
Family pedigree. Affected individuals are marked as black squares and the carriers of genetic variation in *PRPF8* are marked by black dot.

**FIGURE 3:**
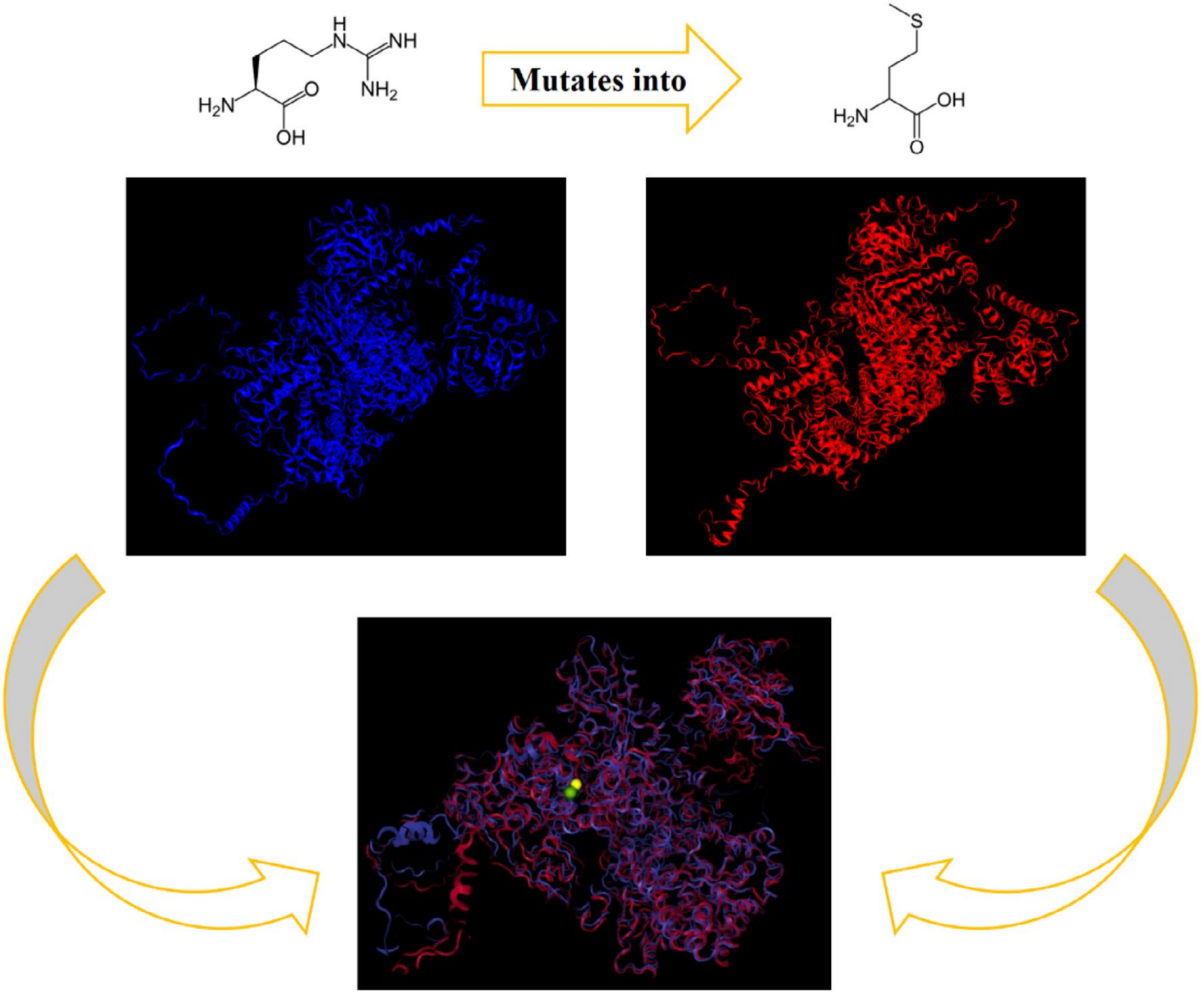
Protein modeling results: Here, we examined a change of the amino acid from arginine to methionine at position 86. The wild‐type protein is shown in blue on the left, while the mutated protein is displayed in red on the right. At the bottom, the superimposed states of the two proteins are presented, revealing a root‐mean‐square deviation (RMSD) of 9.727 Å when comparing these two structures. The two spheres, one yellow and one green, indicate position 86 in the two proteins. The protein images were obtained from https://310.ai/copilot.

The ES was initially performed on the older sister, revealing a variant of unknown significance (VUS) was identified in the *PRPF8* gene (c.257G>T, p.R86M; NM_006445.4). To validate the identified *PRPF8* variant, Sanger sequencing was conducted, confirming homozygosity for the *PRPF8* c.257G>T variant, while the parents were found to be heterozygous carriers. The *PRPF8* homozygous variants were also identified in the similarly affected younger sister. Further investigation of the family history and pedigree revealed that a 31‐year‐old maternal aunt (Figure 1) and a now‐deceased 65‐year‐old maternal great‐aunt exhibited similar features (Figure 2). PCR amplification and Sanger sequencing confirmed that the maternal aunt was also homozygous for the *PRPF8* c.257G>T variant, while her healthy siblings were either normal homozygous or heterozygous, as illustrated in the pedigree (Table 1, Figure 3).

**TABLE 1:** Clinical findings and detailed phenotype of the family members.

*PRPF8* (−) means: c.257G>T p.R86M	Proband −/−	Affected sister −/−	Affected aunt −/−	Father +/−	Mother +/−	Healthy aunt +/+	Healthy uncle +/−
Age (years)	17	12	31	43	37	18	27
Growth and build							
Birthweight	SGA (< 10%)	SGA (< 10%)	SGA (< 10%)	N	N	N	N
Present height	Short (Proportionate)	Short (Proportionate)	Short (Proportionate)	N	N	N	N
Present weight	Underweight	Underweight	Underweight	N	N	N	N
Present stature	Short	Short	Short	N	N	N	N
Head and neck							
Microcephaly	+, < 3 percentile (51.5 cm)	+, < 3 percentile (50.5 cm)	+, < 3 percentile (52 cm)	N	N	N	N
Arched eyebrows	+	+	−	N	N	N	N
Eyes	Strabismus (esotropia)	Strabismus (esotropia)	Strabismus (esotropia)	N	N	N	N
Visual acuity	N	N	N	N	N	N	N
Eyelashes	Long (Ciliary trichomegaly)	Long (Ciliary trichomegaly)	Long (Ciliary trichomegaly)	N	N	N	N
Ears	Low set	Low set	Low set	N	N	N	N
Hearing	N	N	N	N	N	N	N
Nasal root and tip	Convex and Beaked	Convex and Beaked	Convex and Beaked	N	N	N	N
Teeth	Widely spaced	Widely spaced	Widely spaced	N	N	N	N
Neurologic							
Sleep dysregulation	+	+	+	N	N	N	N
Spasticity	+	+	+	N	N	N	N
Touch hypersensitivity	+	−	+	N	N	N	N
Deep tendon reflexes (DTR)	+3 Clonus: − Babinski: −	+3 Clonus: − Babinski: −	+3 Clonus: − Babinski: −	N	N	N	N
Gross motor delay	+	+	+	N	N	N	N
Hypotonia	−	−	−	−	−	−	−
Neuropsychiatric							
ID/LD^a^	Mild (IQ 50–70)	Mild (IQ 50–70)	Mild (IQ 50–70)	N	N	N	N
Language delay	+	+	+	N	N	N	N
Repetitive behavior	Flapping hands	Flapping hands	Flapping hands	N	N	N	N
Autistic behaviors	+	+	+	N	N	N	N
Poor social relationships	+	+	+	N	N	N	N
Hyperactivity	+	+	+	N	N	N	N
Impulsivity	+	+	+	N	N	N	N
Emotional lability	+	+	+	N	N	N	N
Cranial CT scan	N	N	N/A	−	−	−	−
Cranial MRI	N	N	N	−	−	−	−

^a^ Intellectual disability/Learning disabilities.

The comparison of the two energy‐minimized models revealed that the effect of this missense variant was notable and significant. The RMSD of 9.727 Å indicates a significant deviation in the positions of the residues between the two structures. This suggests that the missense mutation may have caused notable changes in the overall conformation of the protein. A TM score of 0.8867 indicates relatively high decreased similarity among the two models. This change in structure is also observed in the Ramachandran plot by decreasing 0.6% of core residues (i.e., 14 residues out of 2335) using UCLA‐DOE LAB—SAVES v6.1 tools.

In silico study through UniProt and HOPE websites suggests these changes can cause loss of interactions with other molecules.

## 4 Discussion

In this case report, we describe a consanguineous family with multiple affected individuals who possess identical c.257G>T missense variants in *PRPF8* and exhibit similar syndromic features, including NDD. Although *PRPF8* genetic variants are primarily recognized for their association with ocular diseases including RP, our report proves that, akin to a recent study by O'Grady et al. (2022) involving 14 patients with NDD and heterozygous genetic variants in this gene, that homozygous missense variants may also be associated with NDDs and syndromic features. In Table S1, a list of de novo variants in the PRPF8 gene that are included in the https://denovo‐db.gs.washington.edu database which were associated with neurological phenotypes has been compiled. The variants reported in this manuscript have been compared to these variants regarding some prediction tools.

Human diseases associated with *PRPF8* mutations underscore the critical role of pre‐mRNA splicing in maintaining cellular and organismal health. Unraveling the specific mechanisms by which these mutations contribute to each disease holds immense potential for the development of novel diagnostic and therapeutic strategies, ultimately improving treatment outcomes across a wide spectrum of human health challenges (Arzalluz‐Luque et al. 2021; O'Grady et al. 2022). *PRPF8* resides is central to the dynamic spliceosome complex and, through its multifaceted contributions; it safeguards the accurate recognition of splice sites, stabilizes the assembly of the splicing machinery, and participates in the enzymatic steps that precisely splice out introns and join exons together (Wickramasinghe et al. 2015). It plays a critical role in pre‐mRNA splicing as a core component of precatalytic, catalytic, and postcatalytic spliceosomal complexes, contributing to both the major U2‐type spliceosome and the minor U12‐type spliceosome (Bertram et al. 2017; Luo et al. 1999; Schneider et al. 2002; Zhang et al. 2017, 2019). The carboxy‐terminal region of the spliceosomal protein PRPF8, which modulates the RNA helicase Brr2, is a mutation hotspot linked to retinitis pigmentosa‐type 13, although its precise function in human splicing and tissue‐specific mechanisms remains unclear. Extensive molecular, transcriptomic, and proteomic analyses have demonstrated that PRPF8/Brr2 regulation plays a crucial role in the selection of the 5′‐splice sites (5′SS) by spliceosomes (Zhang et al. 2019). Mutations in *PRPF* genes lead to delayed spliceosome assembly, resulting in splicing defects that are transcript‐specific and changes in alternative splicing patterns (Tanackovic et al. 2011; Wickramasinghe et al. 2015). Tanackovic et al. (2011) concluded that RP is a systemic splicing disorder, though the retina is particularly vulnerable due to its higher splicing activity compared to other tissues. In other words, research indicates that small nuclear RNAs (snRNAs) are expressed at significantly higher levels in the retina, suggesting that this tissue has a greater demand for these factors. This increased demand makes the retina more sensitive to mutations that slightly decrease the amount or function of splicing factors compared to other tissues with lower demands. Therefore, it can be concluded that the retina has a lower threshold for expressing phenotypes associated with variations in splicing factors. This meticulous process ensures the generation of error‐free, functional mRNAs, ultimately governing protein synthesis and cellular function.

**FIGURE 4:**
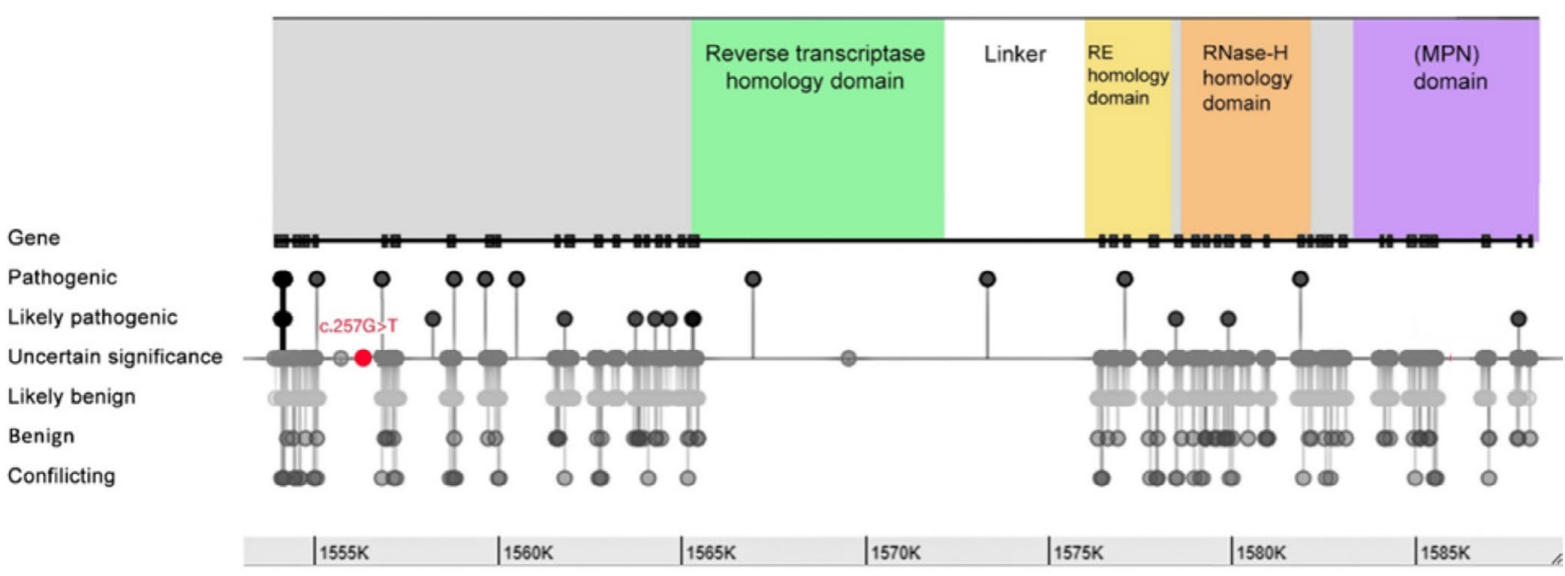
The c.257G>T variant compared to other reported variants, which is located in the MPN domain.

The impact of *PRPF8* mutations on cellular function is not uniform, as a specific domain plays a crucial role (Figure 4). While direct predictions remain complex, understanding the overarching functions of different domains can provide valuable insights (Arzalluz‐Luque et al. 2021). The N‐terminal region of *PRPF8* interacts with various spliceosome components, and mutations in this region could disrupt the formation and function of the complex, leading to impaired splicing initiation. Another key region is the zinc finger domain, which is responsible for recognizing specific RNA motifs at splice sites. Mutations within this domain may hinder accurate splice site selection, resulting in mis‐splicing events and dysfunctional mRNA transcripts. The WD40 repeat region functions as a protein–protein interaction hub, stabilizing the spliceosome structure and facilitating communication between its components; mutations in this region could destabilize the complex, affecting its overall efficiency and accuracy during splicing. The C‐terminal region of *PRPF8* directly contributes to the catalytic steps of splicing and interacts with other splicing factors. Mutations in this region can directly affect the enzymatic activity of the spliceosome, leading to errors in intron removal and exon ligation, ultimately generating nonfunctional mRNAs (Arzalluz‐Luque et al. 2021; Grainger and Beggs 2005).

Despite the recognized role of *PRPF8* in splicing and its association with ocular disorders, particularly the progressive vision loss associated with adRP, recent research paints a more complex, clinically and genetically heterogeneous picture (Martínez‐Gimeno et al. 2003). One area of emerging interest is *PRPF8*′s role in hematological malignancies. Studies suggest that *PRPF8* mutations can contribute to the development of blood cancers, such as acute myeloid leukemia (AML) and myelodysplastic syndromes. These mutations disrupt pre‐mRNA splicing in hematopoietic stem cells, the precursors to various blood cell types. This disruption can lead to uncontrolled proliferation and impaired differentiation of these stem cells, ultimately promoting the development of leukemia and other blood cancers (Kurtovic‐Kozaric et al. 2015; Visconte et al. 2019). In addition to blood cancers, mutations in the gene *PRPF8* are also associated with NDDs. Emerging evidence suggests *PRPF8* plays a role in conditions like autism spectrum disorder ASD and ID. The O'Grady study evaluated 14 NDD patients with primary clinical features including ID, developmental delays, or epilepsy. Some also exhibited additional symptoms like short stature, movement problems, heart defects, and dysmorphic facial features. The *PRPF8* mutations founded in O'Grady et al. study were *de novo*, missense, and loss of function with damaging effects on the protein. While mutations in *PRPF8* were previously linked to vision problems, only one person in this study had RP. Based on these findings, the researchers proposed a new syndrome called *PRPF8*‐associated NDD. While the authors acknowledge that some *PRPF8* mutations might need further investigation, the evidence clearly pointed to *PRPF8* playing an etiologic role in neurodevelopmental disorders (O'Grady et al. 2022).

Consistent with the clinical findings in patients with heterozygous mutations in the *PRPF8* gene, our study demonstrated that patients exhibiting similar features also harbor *PRPF8* mutations that segregate in trans, homozygous (autosomal recessive) configuration. The specific mechanisms remain under investigation, but with a focus on the potential for disruption of splicing in genes crucial for brain development and function (Li et al. 2023). This could lead to abnormal neuronal migration, synaptic dysfunction, and impaired neurotransmitter signaling, all of which functionally contribute to the characteristic features of NDDs. Further research into the intricate structure, function, and disease associations of *PRPF8* holds immense promise for advancing our understanding of gene expression and regulation, potentially paving the way for novel diagnostic tools and therapeutic strategies (O'Grady et al. 2022). Segregation data (PP1) and allelic data (PM3) are compatible with these cases; by applying these two items manually in franklin, Varsome, and Clingen databases, this variant turns likely pathogenic.

Epigenetic modifications, including DNA methylation (DNAm), are often related to environmental exposures and are increasingly recognized as key processes in the pathogenesis of disease (Domingo‐Relloso et al. 2022). An epigenome‐wide study of DNA methylation profiles and lung function among American Indians investigation in 2022 showed that PRPF8, along with EGFR and MAPK1 genes, were the most connected nodes in the protein–protein interaction network (Domingo‐Relloso et al. 2022). The PRPF8 gene encodes a crucial component of the spliceosome, which is integral to RNA splicing and processing. Given its role in transcriptional regulation and potential influence on chromatin organization, it is conceivable that alterations in PRPF8 function could have cascading effects on epigenetic mechanisms, including DNA methylation (Matera and Wang 2014; Will and Lührmann 2011). While our study did not directly investigate the methylation profile, we acknowledge the importance of this aspect. The homozygous PRPF8 variant may disrupt splicing patterns, potentially affecting the expression of key regulatory genes involved in neurodevelopment, some of which may possess methylation‐sensitive promoters. Furthermore, splicing factors have been implicated in the regulation of chromatin modifiers, which could lead to broader epigenetic dysregulation (Luco et al. 2011; Naftelberg et al. 2015). The PRPF8 p.H2309P mutation has been shown to enhance the usage of cryptic splice sites, particularly in retinal‐specific and ciliary transcripts, and the PRPF8 p.H2309P mutation causes dispersion of nuclear splicing speckles in retinal cells, so PRPF8 mutations affect spliceosome activation and efficiency (Atkinson et al. 2024; Mayerle and Guthrie 2016).

Spliceosomal dysfunction resulting from PRPF8 variants may indirectly influence the methylation landscape by altering the splicing and expression of genes involved in epigenetic regulation. Subsequent studies investigating the methylation profile of affected individuals, particularly utilizing tools such as the Illumina Infinium HumanMethylation EPIC array or whole‐genome bisulfite sequencing, could provide valuable insights into potential methylation signatures associated with PRPF8 mutations (Fortin et al. 2017; Suzuki et al. 2018). Such analyses may elucidate whether methylation abnormalities contribute to the neurodevelopmental phenotype observed in our study.

The results of the protein modeling also provided further evidence supporting the pathogenicity of this variant. The findings indicated that the introduced missense change has a significant impact on the structure. Since this protein interacts with several other proteins, this structural alteration could affect the overall function of the splicing complex and explain the variability of symptoms in affected individuals. This effect could be investigated through RNA‐seq studies; however, due to the inability to sample the target tissue (which likely required a higher level of this complex), we are unable to conduct such an analysis.

To assess the probability of whether the presence of this variant in a homozygous state and the occurrence of the disease are coincidental, the following method was employed. By utilizing the status affected individuals and their siblings for calculation, whose sequencing results are known, the following outcomes were obtained: Since both parents are heterozygous, the allele frequencies of the mutant and wild‐types are equal, each being 0.5 (*p* + *q* = 1; *p* = *q*). Consequently, the probability of a child being homozygous for the identified variant is 25%, while the probabilities of being heterozygous or homozygous for the wild‐type are 75%. According to the pedigree (Figure 1), the most probable inheritance pattern of the disease is autosomal recessive, which indicates a 25% probability of the child being affected and a 75% probability of being unaffected. The likelihood of this scenario occurring by chance is calculated by multiplying the probabilities of the aforementioned states for 8 children, in probability of the status of 3 out of 8, resulting in a very low probability {83 ((0.75)^5^(0.25)^3^) ((0.75)^5^(0.25)^3^) = 0.00077}. This suggests that the chance occurrence of this association is exceedingly rare. Therefore, this probability, alongside other evidence of the pathogenicity of this variant, renders it more plausible.

The homozygous *PRPF8* c.257G>T (p.R86M) variant identified in this study represents a novel missense alteration within a functional domain of the PRPF8 protein. While our findings suggest a potential association between this variant and the observed neurodevelopmental phenotype in the affected family, the absence of functional data precludes definitive conclusions regarding causality (MacArthur et al. 2014). Notably, the GnomAD database reports several homozygous variants nearby in apparently unaffected individuals, indicating that not all variants within this domain are necessarily pathogenic (Karczewski et al. 2020). These observations emphasize the critical need for functional studies to elucidate the impact of the p.R86M substitution on PRPF8 function and its potential contribution to NDD (Richards et al. 2015). Such investigations would provide valuable insights into the variant's pathogenicity and its role in the observed clinical presentation.

The *PRPF8* c.257G>T (p.R86M) variant represents a missense substitution within a conserved functional domain of the protein. Missense variants are less frequently associated with autosomal recessive inheritance compared to nonsense or frameshift variants, which typically result in loss of function (Kurtovic‐Kozaric et al. 2015). It is conceivable that the p.R86M variant disrupts PRPF8 function, potentially leading to impaired splicing activity. PRPF8 plays a crucial role in the spliceosome, particularly in the regulation of Brr2 helicase activity, which is essential for splice site selection and maintaining splicing fidelity (Atkinson et al. 2024). In our study, heterozygous parents carrying the p.R86M variant were asymptomatic, consistent with autosomal recessive inheritance. No neurodevelopmental or behavioral symptoms were observed upon clinical evaluation. This observation suggests that heterozygous carriers retain sufficient PRPF8 activity to avoid overt clinical manifestations, though subclinical effects cannot be excluded and warrant further exploration. However, functional studies are necessary to determine whether this variant causes loss of function, dominant‐negative effects, or gain of function. Previous research has shown that PRPF8 mutations can lead to mis‐splicing in various tissues, with retina‐specific genes being particularly sensitive to reduced PRPF8 levels (Malinová et al. 2017). To fully elucidate the impact of the p.R86M variant, future studies should investigate its effects on spliceosome assembly, splicing efficiency, and potential tissue‐specific consequences.

The zebrafish model with PRPF8 knockout demonstrated extensive neural degeneration, characterized by widespread apoptosis in the central nervous system, including both the brain and spinal cord (Keightley et al. 2013). This observation led researchers to propose that the absence of PRPF8 protein is a critical factor in triggering neuronal cell death (Keightley et al. 2013). Furthermore, population‐level genetic data indicate that PRPF8 exhibits a high degree of intolerance to variation, as evidenced by its elevated constraint metrics (missense *z*‐score of 11.34 and pLI = 1; o/e = 0.21 [0.17–0.26]; https://gnomad.broadinstitute.org) (Karczewski et al. 2020).

In conclusion, our report suggests a possible novel pattern of inheritance for *PRPF8*‐related genetic variants that result in craniofacial syndromic features, microcephaly, and NDDs, including autism and ID. To the best of our knowledge, this report is the first report discussing the probability that two identical homozygous missense variants within *PRPF8* may have pathogenic effects contributing to NDDs, including autism and ID. Further studies of animal models will be essential for understanding the adverse effects on function at the brain level and the specific neurodevelopmental mechanisms involved.

## Author Contributions

R.J.E.: Reviewing the literature; organizing the table; writing the original draft. M.R.M.: Initial assessment of the case; ordering the genetic testing; interpreting the results; editing the original draft. F.M., A.S., and S.F.: Confirming genetic analysis; reviewing; editing the draft. A.S. and M.R.S.: literature review; editing the draft; protein modeling; mathematical calculation; and revision. M.E.S.L. and M.B.T.: Conception; final approval; writing and editing the draft.

## Consent

The patient's family has signed informed consent regarding publishing their data and their pictures. The manuscript is a retrospective case report that does not require ethics committee approval at that institution.

## Conflicts of Interest

The authors declare no conflicts of interest.

## Supporting information


**Table S1** A compilation of de novo variants in the *PRPF8* gene associated with neurological phenotypes, as documented in the https://denovo‐db.gs.washington.edu database, is presented. Variant reported in this manuscript was compared to these de novo variants using various predictive tools.

## Data Availability

The datasets generated during this study are available only on reasonable request.
