# Supplementary material for: Reporting a Homozygous Case of Neurodevelopmental Disorder Associated With a Novel PRPF8 Variant
Source: Mol Genet Genomic Med. 2025 Mar 11;13(3):e70084. doi: 10.1002/mgg3.70084 (PMC11894437; doi:10.1002/mgg3.70084)
Supplement: Supplementary file 1 — Table S1 A compilation of de novo variants in the PRPF8 gene associated with neurological phenotypes, as documented in the https://denovo‐db.gs.washington.edu database, is presented. Variant reported in this manuscript was compared to these de novo variants using various predictive tools. [file MGG3-13-e70084-s001.docx]

Supplementary Table 1. A compilation of de novo variants in the *PRPF8* gene associated with neurological phenotypes, as documented in the https://denovo-db.gs.washington.edu database, is presented. Variant reported in this manuscript was compared to these de novo variants using various predictive tools.

| Mutation | Phenotype | Frequency | ACMG classification | CADD | | SIFT | PolyPhen | REVEL | DANN | PrimateAI | SpliceAI | GERP | Aggregated Prediction | Publication |
| --- | --- | --- | --- | --- | --- | --- | --- | --- | --- | --- | --- | --- | --- | --- |
| c.257G>T  p.R86M | **ID, microcephaly** | **0** | **VUS** | **31** | **Deleterious (Supporting) (0)** | | **NA** | **Uncertain (0.62)** | **Deleterious (0.96)** | **Deleterious (Moderate) (0.95)** | **-** | **Uncertain (5.06)** | **Uncertain (0.68)** | **This manuscript** |
| c.1003C>T  p.P335S | Autism | 0.000008 | VUS | 19.761 | Uncertain (0.013) | | Uncertain (0.54) | Deleterious (Moderate) (0.83) | Deleterious (1) | Deleterious (Moderate) (0.88) | - | Uncertain (5.65) | deleterious | PMID: 25363768 |
| c.1984+1G>C | Autism | 0 | Likely pathogenic | 24.601 | NA | | NA | NA | Deleterious (1) | NA | Splice-Altering / strong (0.91) | Uncertain (5.94) | Deleterious (0.8) | PMID: 28263302 |
| c.1897G>A  p.G633S | Autism | 0 | VUS | 21.701 | Benign (Moderate) (0.44) | | Benign (Supporting) (0.02) | Uncertain (0.38) | Deleterious (1) | Uncertain (0.77) | - | Uncertain (5.94) | Uncertain (0.47) | PMID: 28263302 |
| c.6055C>T  p.P2019S | Developmental Disorder | 0 | VUS | 33 | Uncertain (0.021) | | NA | Uncertain (0.53) | Deleterious (1) | Deleterious (Moderate) (0.87) | - | Uncertain (6.17) | Uncertain (0.6) | PMID: 28135719 |
| c.5539G>A  p.A1847T | Developmental Disorder | 0 | VUS | 35 | Uncertain (0.004) | | NA | Deleterious (Supporting) (0.68) | Deleterious (1) | Deleterious (Supporting) (0.79) | - | Uncertain (5.56) | Deleterious (0.71) | PMID: **28135719** |
